# A systematic scanning method to locate cryptic terrestrial species

**DOI:** 10.1016/j.mex.2024.103038

**Published:** 2024-11-07

**Authors:** Rachel Findlay-Robinson, Davina L. Hill

**Affiliations:** a Institute of Science and Environment, University of Cumbria, Ambleside, Cumbria, LA22 9BB UK; b Graduate School, College of Medical, Veterinary and Life Sciences, University of Glasgow, Glasgow, G12 8QQ, UK; c School of Biodiversity, One Health and Veterinary Medicine, College of Medical, Veterinary and Life Sciences, University of Glasgow, Glasgow, G12 8QQ, UK; d School of Animal, Plant and Environmental Sciences, University of the Witwatersrand, Johannesburg, Private Bag 3, Wits 2050, South Africa

**Keywords:** PIT tag, Hibernation, Crypsis, A systematic search protocol for locating cryptic, ground-dwelling PIT-tagged animals

## Abstract

When studying wild animals, consideration must be given to potential detrimental effects of the study technique, particularly if techniques may affect behaviour or energy expenditure. Many small terrestrial species occupy cryptic habitats, the characteristics and locations of which may be poorly understood. To study these habitats, researchers must be able to locate them, but must also consider the potential for disturbance of the organisms and the impacts this may have. Here, we developed and tested a novel, non-invasive method of locating the cryptic hibernation nests of passive integrated transponder (PIT) tagged hazel dormice *Muscardinus avellanarius*. The use of a powerful PIT tag scanner combined with a systematic search technique resulted in the location of nine wild hibernating dormice. Camera trap recordings indicated no external dormouse activity following detections, indicating minimal disturbance. In addition, eleven PIT tags no longer inside a dormouse were detected on the forest floor during searches. This study demonstrates a non-invasive alternative to techniques such as radio-collaring for small mammals, and highlights potential uses of PIT tags in research beyond identification of individuals, particularly in understanding fine-scale habitat selection.

• A systematic search method enabled location of cryptic terrestrial species
• The use of PIT tags allows detection with minimal disturbance

## Graphical abstract

**Figure:**
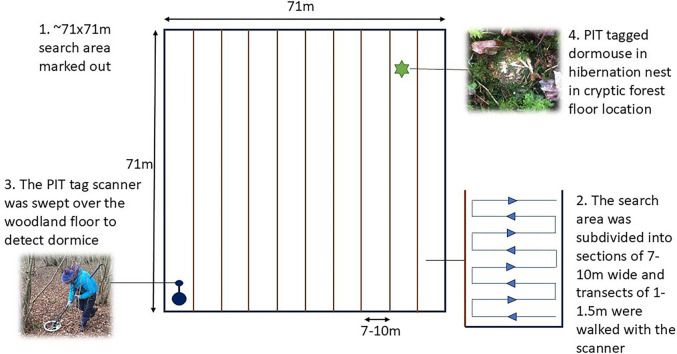

Specifications table

**Table:** 

Subject area:	Agricultural and Biological Sciences
More specific subject area:	*Zoology*
Name of your method:	A systematic search protocol for locating cryptic, ground-dwelling PIT-tagged animals.
Name and reference of original method:	Salazar RD, Montgomery RA, Thresher SE, Macdonald DW. Mapping the relative probability of common toad occurrence in terrestrial lowland farm habitat in the United Kingdom. PLOS ONE. 2016 Feb 3;11(2):e0148269.
Resource availability:	Equipment used by the authors: Scanning array https://www.biomark.com/product/hpr-plus-handheld-pit-tag-reader/ https://www.biomark.com/product/bp-plus-portable-antenna/ https://www.peddymark.com/pet-microchip-products/mini-microchips/ Please note other microchip and scanner systems are available, but not all microchips will work with all scanning systems

## Background

When studying wild animals, consideration must be given to potential detrimental effects of the study technique. This is particularly relevant to techniques that may affect behaviour or energy expenditure, which may subsequently reduce individual fitness and/or survival. Small or cryptic species can be located using visual or hand searching [1], or radio-transmitters (e.g. [2,3]), although this requires trapping or otherwise locating individuals first); however, these methods risk causing disturbance to individuals. This may be detrimental where even non-tactile human disturbance may induce unnecessary energy expenditure, such as in hibernating animals [4]. Additionally, visual or hand searching is demanding in terms of both time and energy, may require significant experience, and may not successfully locate animals that are sufficiently concealed, such as under thick layers of vegetation. Systematic searches used in conjunction with passive integrated transponder (PIT) tag scanning technology may increase the success of locating such cryptic organisms [5]. Here, we aimed to validate a non-invasive method of locating wild hibernating hazel dormice in already PIT tagged populations using a systematic search method with a powerful PIT tag reader. Hazel dormice are a threatened UK species which hibernate in cryptic locations at ground level. Searches for hazel dormouse hibernation nests are usually carried out by hand, and may take between four and seven hours for an experienced surveyor to search 1 ha of land (People's Trust for Endangered Species, pers. comms; [6]) depending on the ground conditions. Searches for hazel dormouse hibernation nests often form part of protected species mitigation conditions for planning and development licences in the UK.

## Method validation

**Equipment used:**

- HPR Plus reader (Biomark Inc., Idaho)
- BP Plus portable antenna (Biomark Inc., Idaho)
- 1.4 × 8 mm International Standards Organization (ISO) microchips (Peddymark Ltd., Bishops Stortford, UK)
- Garmin Etrex 10 Global Positioning System (GPS) unit (Garmin Ltd, Kansas, USA)
- Biodegradable flagging tape

The HPR Plus reader and BP Plus portable antenna will hereafter be referred to as “the scanning array”.

**Fig. 1:**
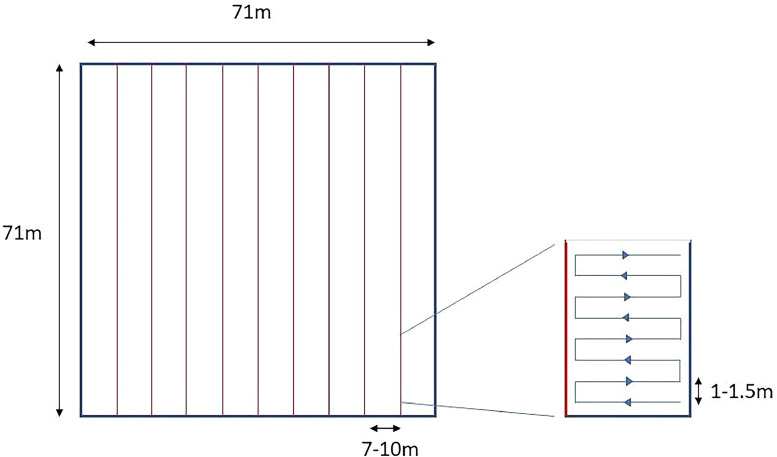
Schematic of search area layout used in blind trials and a study to locate PIT-tagged hibernating dormice using a PIT tag scanning array. A 5000m^2^ search area was delimited and divided into smaller sections approximately 71 m x 7–10m. These smaller sections were walked as repeated transects, each approximately 1–1.5 m from the previous.

Prior to use in the field, the effectiveness of the proposed technique was tested using a validation study. During this study, a Peddymark Mini ISO microchip with dimensions 1.4 × 8 mm (hereafter referred to as a PIT tag) was inserted into a piece of cooled cooked sausage and placed inside woven grass to simulate a hibernating dormouse in a nest (hereafter referred to as a simulated dormouse). The validation study consisted of two stages:

(1) non-blind trials: the simulated dormouse was placed in a known location on four different substrates representative of potential hibernation locations (bare ground, under a thin layer of soil, under rocks or under a log). The maximum distance at which the scanning array could detect the simulated dormouse in each substrate was recorded.
(2) blind trials: a 5000m^2^ search area (∼71 m x 71 m) was marked out with flagging tape and subdivided into ∼71 m x 10–15 m areas (depending on habitat type; see below) to shorten the length of each transect. This was intended to increase scanning accuracy, compared to longer transects. The field assistant placed the simulated dormouse in a cryptic position within the search area whilst the operator was absent, and marked the location on a GPS device. The operator systematically searched each of the subdivided areas by slowly walking parallel transects along the width of the section whilst sweeping the ground with the scanning array. Each transect line was approximately 1–1.5 m from the previous line, thus allowing a small margin of overlap between sweeping areas (Fig. 1). This trial was carried out 15 times, five times in each of three habitat classifications (Table S1). Searches continued until (i) the simulated dormouse was located, or (ii) the entire search area had been scanned if the simulated dormouse was not located.

The non-blind trial was performed at Gaitbarrows National Nature Reserve, Carnforth, Lancashire, UK, and the blind trials were carried out in several different woodland habitats (Table S1) around Cumbria, UK. Search areas in the blind trials were classified into three habitats, described as (1) mature woodland with little understorey [although bracken was present during some searches], hereafter referred to as “mature habitat”; (2) mid-aged woodland with understorey (referred to as “mid habitat”); (3) young woodland with understorey (referred to as “young habitat”). All search areas were chosen to represent habitats known to support dormice, but that were known not to have existing dormouse populations to avoid disturbance.

**Fig. 2:**
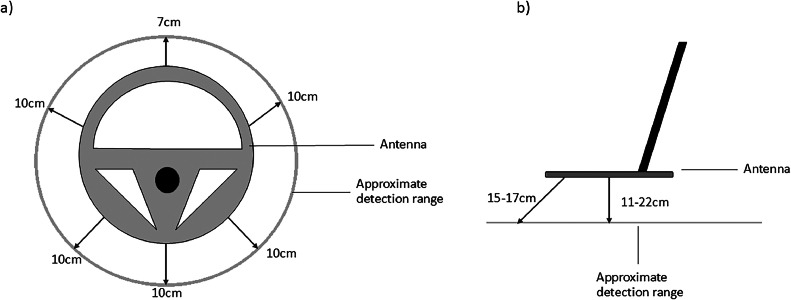
Distances at which a HPR Plus reader with a BP Plus Portable Antenna (Biomark Inc. Idaho, USA) was able to detect a Peddymark Mini ISO microchip (Peddymark Ltd., Bishops Stortford, UK) with dimensions 1.4 mm x 8mm. (a) shows horizontal detection distances, i.e. where the antenna is flat on the floor with the microchip horizontal to the antenna, and (b) shows vertical detection distances, i.e. where the antenna is above the microchip.

Non-blind trials showed that the detection distance for PIT tags varied from 7 to 22 cm (mean = 12.92 ± 5.07 standard deviation (SD)), depending on PIT tag orientation and the part of the antenna detecting the tag (Fig. 2). The outer edges of the antenna detected the tags at greater distances than the centre of the antenna. Due to this, the antenna was kept in motion through a side-to-side sweeping action during the blind trials to increase the likelihood of all areas of the detector passing near a PIT tag. The substrate that the PIT tag was placed in did not affect the read distance (One-way Analysis Of Variance (ANOVA), *F* = 0.17, df = 3, *p* = 0.91).

**Fig. 3:**
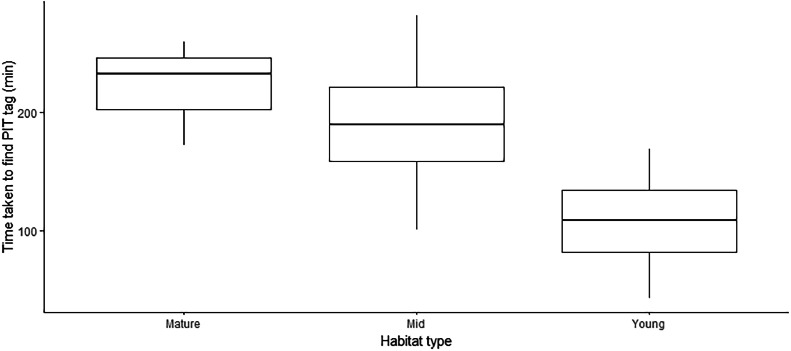
Time taken to find PIT tags in three different habitat types during blind search trials. Where PIT tags were successfully located, there were no significant differences in the time taken dependent on habitat type. The mean time taken to find a microchip in successful trials was 168 ± 73 min. N (mature) = 3, n (mid) = 4, n (young) = 4 trials. Horizontal lines within boxes indicate the median. Lower and upper box edges indicate 25th and 75th percentiles, whilst whiskers indicate the overall spread of the data.

In the blind trial, the simulated dormouse was located in 73% of cases. The mean time taken to locate the simulated dormouse was 168 ± 73 min (mean ± SD, Table S1). Habitat type did not affect likelihood of detection (Binomial Generalised Linear Model, *p* = 0.84) and, in successful trials, there was no difference in the time taken to locate a PIT tag between different habitat types (One-way ANOVA, *F* = 0.10, df = 2, *p* = 0.90, Fig. 3). The results of the blind trial were used to fine-tune the scanning technique and inform decisions as to where to concentrate search effort when the technique was used in a study with wild dormice described in [7]. For example, it was found that the presence of bracken or thick brambles increased the difficulty of accurate scanning and decreased the likelihood of locating the simulated dormouse. Scanning in these areas was considered to increase the likelihood of accidentally harming a hibernating dormouse through not being able to scan the ground before stepping on it. These areas were therefore avoided during the fieldwork study described in [7].

## Use of technique part of a field study with wild dormice

This technique was used to locate hazel dormouse hibernation nests as part of a study into microclimates surrounding nests [7]. During the study, nine hibernating dormice and seven loose PIT tags (i.e. PIT tags that were lost. e.g. through predation of dormice, or tags emigrating from the dormouse before the insertion site had healed [8]) were located using the systematic search technique (Table S2). One sealed hibernation nest was also found visually, despite no PIT tag being detected.

## Limitations

The PIT tag scanning technique described here was generally successful, although some limitations were noted. Thick vegetation and undergrowth were found to increase the difficulty of manoeuvring the scanner, and also increased likelihood of disturbance or injury to a dormouse. The scanning array used was relatively heavy and cumbersome; smaller arrays are commercially available, but tend to have lower detection distances. Detection distances increase when used with larger PIT tags, but consideration needs to be given to the effect on the animal. The internal battery of the scanner lasts for approximately 4 h in the most frequent read-mode. External batteries are available, but also need to be carried by the operator, increasing the weight of the equipment.

Although the scanning technique itself is non-invasive, insertion of PIT tags is invasive. If animals are being tagged exclusively for purposes of relocation, it should be considered whether additional data can be gathered to justify the use of an invasive technique. In addition to necessary permits being required, suitable training must be provided for researchers involved in PIT tagging to minimise effects of tagging on the study organisms. Where the study species is rare or protected, acquiring sufficient experience prior to the study can be difficult.

Depending on the size and model of the scanning array used, the initial outlay for this technique may be expensive; the HPR Plus reader and BP Portable antenna currently retail at $3670 and $2150 USD respectively. However, basic PIT tags, such as those used here, are relatively inexpensive (<$5 USD each).

## Ethics statements

All research in dormouse habitats was carried out under project licences granted to R. Findlay-Robinson (Natural England Science Education and Conservation Schedule 5, licence number 2020–44,498-SCI-SCI; Natural Resources Wales licence number S087420/1) and with the permission of the landowners. Ethical approval was granted by the University of Cumbria Research Ethics Panel (Reference 19/05).

## CRediT authorship contribution statement

**Rachel Findlay-Robinson:** Conceptualization, Methodology, Validation, Writing – original draft, Writing – review & editing, Funding acquisition. **Davina L. Hill:** Conceptualization, Methodology, Writing – review & editing, Funding acquisition, Supervision.

## Declaration of competing interest

The authors declare that they have no known competing financial interests or personal relationships that could have appeared to influence the work reported in this paper.

## Data Availability

Data are provided in the supplementary materials

## Appendix Supplementary materials

Image, application 1
