# Supplementary material for: A systematic scanning method to locate cryptic terrestrial species
Source: MethodsX. 2024 Nov 7;13:103038. doi: 10.1016/j.mex.2024.103038 (PMC11609314; doi:10.1016/j.mex.2024.103038)
Supplement: Supplementary file 1 [file mmc1.docx]

**Supplementary material *and/or* additional information [OPTIONAL]**

**Table S1. Time taken in three habitat types to locate a simulated hibernating dormouse containing a PIT tag with a PIT tag scanner (Biomark HPR with BP antenna) using a systematic search technique. NA indicates that the simulated dormouse was not found. Habitat types: “Young” = Young woodland with understorey, “Mid” = mid-aged woodland with understorey, “Mature” = mature woodland with little understorey.**

| **Location** | **Habitat type** | **PIT tag found?** | **Time taken to locate PIT tag (min)** |
| --- | --- | --- | --- |
| Gaitbarrows National Nature Reserve | Young | N | NA |
| Brundholme Woods | Young | Y | 95 |
| Brundholme Woods | Young | Y | 123 |
| Brundholme Woods | Young | Y | 169 |
| Nannygate Woods | Mature | Y | 172 |
| Nannygate Woods | Mature | Y | 260 |
| Banerigg Woods | Mid | N | NA |
| Banerigg Woods | Mid | Y | 201 |
| Latrigg Woods | Mature | N | NA |
| Latrigg Woods | Mature | Y | 233 |
| Greenbank Woods | Mid | N | NA |
| Greenbank Woods | Mid | Y | 282 |
| Greenbank Woods | Mid | Y | 101 |
| Greenbank Woods | Mid | Y | 178 |
| Brundholme Woods | Young | Y | 43 |

**Table S2. Details of searched for hibernating dormice during the field study described in (7).**

| **Study site** | **Month and year of search** | **Search area identity** | **No. chips detected** | **Comments** |
| --- | --- | --- | --- | --- |
| Bradfield | February 2020 | 1 | 0 |  |
| Bradfield | February 2020 | 2 | 2 |  |
| Bradfield | February 2020 | 3 | 1 | Loose chip |
| Bradfield | February 2020 | 4 | 0 |  |
| Bradfield | February 2020 | 5 | 0 |  |
| Bradfield | February 2020 | 6 | 1 | Loose chip |
| Bontuchel | February 2020 | 1 | 0 |  |
| Bontuchel | February 2020 | 2 | 2 | 1 of these was a sealed hibernation nest found visually but no chip detected. |
| Bontuchel | February 2020 | 3 | 1 | Loose chip |
| Bontuchel | February 2020 | 4 | 1 | Loose chip |
| Bontuchel | February 2020 | 5 | 1 | Dormouse under leaf litter not in a hibernation nest |
| Bontuchel | February 2020 | 6 | 0 |  |
| Bradfield | November 2020 | 1 | 0 |  |
| Bradfield | November 2020 | 2 | 0 |  |
| Bradfield | November 2020 | 3 | 1 |  |
| Bradfield | November 2020 | 4 | 2 |  |
| Bradfield | November 2020 | 5 | 1 |  |
| Bontuchel | November 2020 | 1 | 2 | Both loose chips |
| Bontuchel | November 2020 | 2 | 0 |  |
| Bontuchel | November 2020 | 3 | 0 |  |
| Bontuchel | November 2020 | 4 | 0 |  |
| Bontuchel | November 2020 | 5 | 1 | Loose chip |
